# Improving Pre-Exposure Prophylaxis Adherence in People at Risk for HIV: Secondary Analysis of a Digital Health Intervention to Enhance User Engagement

**DOI:** 10.2196/66837

**Published:** 2026-03-10

**Authors:** Lisa Hightow-Weidman, Seul-Ki Choi, Ibrahim Yigit, Crissi Rainer, Victoria Phillips, Kathryn Muessig

**Affiliations:** 1 Institute on Digital Health and Innovation, College of Nursing, Florida State University, Innovation Park, Research Building B, 2010 Levy Avenue, RM B3400, Tallahassee, FL, 32310, United States, 1 9199499540; 2 Department of Family and Community Health, College of Nursing, University of Pennsylvania, Philadelphia, PA, United States; 3 Department of Health Policy and Management, Rollins School of Public Health, Emory University, Atlanta, GA, United States

**Keywords:** pre-exposure prophylaxis, PrEP, digital health intervention, DHI, engagement, gamification, applications, user engagement, users, chi-square analysis, adherence, engagement, coaching, costs, HIV, young adults, young individuals, youth, prophylaxis, optimizing, optimization, apps

## Abstract

**Background:**

Although highly effective HIV pre-exposure prophylaxis (PrEP) is available, its usage and adherence among young men who have sex with men and young transgender women remain low, reducing its overall effectiveness. The study included a 3-arm randomized clinical trial of Prepared, Protected, emPowered (P3), a comprehensive PrEP adherence digital health intervention, compared to an enhanced version, P3+, which incorporates in-app adherence coaching.

**Objective:**

This study aims to analyze data from study participants in the P3/P3+ intervention arms to understand how different levels of user engagement with the app’s features were associated with adherence to PrEP as well as the costs of each intervention and their relative cost-effectiveness.

**Methods:**

Descriptive statistics for study variables at baseline were calculated. To examine the differences in intervention engagement and acceptability by arm, independent samples 2-tailed *t* tests for continuous variables and a chi-square analysis for categorical variables were conducted. To examine the effect of arm and engagement categories on PrEP adherence at 3 months, three logistic regression analyses were conducted: (1) the effect of arm on PrEP adherence, (2) the effect of predefined engagement categories (high vs moderate and low) on PrEP adherence, and (3) the interaction effect of arm and predefined engagement categories on PrEP adherence, along with the main effects of arm and predefined engagement categories. The study team calculated the average cost per participant and the incremental cost-effectiveness for PrEP adherence and engagement measures.

**Results:**

A total of 163 participants were randomized to the P3 intervention (82 to the P3 arm and 81 to the P3+ arm). Participants in the P3+ arm earned higher incentives (US $90.6 vs $75.4; *P*=.04), had more app log-ins (96.6 vs 76.1; *P*=.01), used the app on more days (63.3 d vs 53.2 d; *P*=.04), and spent more time in the intervention (378.8 min vs 186.66 min; *P*<.001) compared to those in the P3 arm. There was no significant association between intervention arm and PrEP adherence at 3 months (*P*=.99). Engagement category (high vs moderate or low) was significantly associated with PrEP adherence at 3 months (*P*=.003). The overall average total monthly cost of P3 was US $1118 (SD $305.1). Average total monthly cost per P3 participant was $280 (SD $118.5), with an additional cost for P3+ of $72.

**Conclusions:**

This study highlights the critical role of user engagement in enhancing PrEP adherence among young individuals at high risk for HIV. While the P3+ intervention led to increased engagement, this did not translate into significantly better adherence compared to the standard P3 arm. This, coupled with the increased cost and complexity of P3+ delivery, indicates that further studies are necessary to determine whether this intensified intervention is the appropriate fit.

## Introduction

The introduction of pre-exposure prophylaxis (PrEP) has been a groundbreaking advancement in the drive to eliminate HIV [1]. Despite its efficacy, the overall impact of PrEP is heavily influenced by adherence levels among users [2]. High adherence to PrEP is crucial for its effectiveness in preventing HIV infection, yet PrEP adherence over time is poor, particularly among youth [3,4]. To reap the potential benefits, interventions for young men who have sex with men and young transgender women who are disproportionately impacted by HIV in the United States that can enhance and maintain PrEP adherence over time are needed [5,6].

In recent years, digital health interventions (DHIs) have emerged as promising tools in promoting health behaviors, including medication adherence [7]. These interventions offer innovative ways to engage users, provide information, and support adherence through interactive and personalized digital platforms. Engagement in DHIs has been shown to play a critical role in their efficacy in supporting behavior change across a variety of health domains [8-11]. Research has consistently demonstrated that increased user interaction with digital platforms is associated with improved outcomes, particularly in the context of HIV prevention and care [12-15].

The mechanisms through which engagement enhances efficacy are multifaceted. Active participation in DHIs can lead to increased knowledge, stronger motivation, and better self-management skills among users [16-18]. These interventions often use reminders, motivational messages, and educational content, which help sustain users’ commitment to their health regimens [16,17]. Promotion of engagement through incentives and gamification has also shown promise, though studies documenting their ability to maintain engagement over time are limited [19-22].

The Adolescent Medicine Trials Network for HIV Interventions (ATN) conducted a study using the Prepared, Protected, emPowered (P3) app, a comprehensive PrEP adherence DHI, and its enhanced version, P3+, which incorporates in-app adherence coaching [23]. The P3 app included multiple features aimed at increasing user engagement, including a social wall, daily quests, and medication tracking, as well as both in-app “virtual rewards” in the form of unlocking video series produced specifically for the study and real-world rewards in the form of monetary incentives awarded based on principles of behavioral economics [24-26]. This study provided a rich dataset to explore how digital engagement correlates with PrEP adherence among a young demographic at high risk for HIV. This paper seeks to analyze data from a subset of the P3 study participants—those who were part of the intervention arms using P3 and P3+. The focus is on understanding how different levels of user engagement with the app’s features were associated with adherence to PrEP as well as the costs. This analysis is crucial, as it contributes to optimizing digital health strategies to support sustained PrEP use.

## Methods

### Parent Study

P3 is a comprehensive PrEP adherence app which incorporates a variety of content in multiple formats including articles, videos, quizzes, and a social wall in which participants can share experiences, from success stories to challenges. P3 core components included: social prompts to foster community among participants; medication tracking and adherence support; brain builders, which included goal setting and knowledge building; and the knowledge center. P3+ is an extension of P3, in which participants receive all components in P3 and the addition of in-app adherence coaching using an adapted curriculum based on Next Step Counseling adherence counseling [27]. The study included a 3-arm randomized clinical trial conducted from March 2019 to September 2021 and followed each participant for 6 months. Participants were recruited through the ATN from 9 participant recruitment venues in Bronx, New York; Chicago, Illinois; Atlanta, Georgia; Houston, Texas; Boston, Massachusetts; Philadelphia, Pennsylvania; Tampa, Florida; Charlotte, North Carolina; and Chapel Hill, North Carolina. Recruitment methods included in-person, venue-based (including recruitment from local PrEP clinics or providers) and web-based recruitment on social media platforms. Eligible participants were (1) aged 16 to 24 years; (2) assigned male sex at birth; (3) reported sex with or intentions to have sex with men or transgender women; (4) had reliable daily access to an Android or iOS smartphone with a data plan; (5) able to speak and read English; (6) self-reported HIV-negative; and (7) were either not currently on PrEP but planned to initiate in the next 7 days and had an active PrEP prescription (prescription confirmed by study staff) or currently on PrEP and had an active PrEP prescription (prescription confirmed by study staff). Additional information about the P3 study has been previously published [23]. The original study had a baseline survey with 3- and 6-month follow-up. However, this secondary analysis only included those participants randomized to the 2 intervention arms (n=163) and data from baseline (demographic characteristics), 3-month follow-up (PrEP adherence and acceptability), and app data (engagement).

### Measures

#### PrEP Adherence

The study team developed a composite PrEP adherence score to balance data accuracy and completeness across participants by combining biological and self-reported measures: (1) objective estimates of drug levels from dried blood spots, specifically tenofovir-diphosphate (TFV-DP) and (2) self-reported PrEP adherence in the past month. The composite score first used the participants’ TFV-DP levels in the blood consistent with more than 4 pills per week considered as adherent (0=“nonadherent” and 1=“adherent”). For those without TFV-DP measurements (ie, missing laboratory data point), self-reported PrEP adherence from the 3-month survey was used, as prior studies have demonstrated a strong correlation between self-report and biomarker-based adherence measures [28,29]. Self-reported PrEP adherence in the past month was assessed with a single question: “In the last month, what percent of the time did you take your PrEP as prescribed [once a day]?” with response options ranging from 0% (none of the time) to 100% (all of the time). Following previous research, participants without a TFV-DP measurement but with self-reported adherence were considered adherent if they reported taking PrEP 60% or more of the time in the past month [2]. Among participants with both TFV-DP and self-reported adherence data (n=97), the 2 measures demonstrated moderate agreement (Cohen κ=0.405), with no significant difference detected by the McNemar test (test statistic=2.25 *P*=.14), supporting the reliability of using both measures to assess adherence.

Although in-app daily pill-taking data were collected, these data were not used as the primary adherence measure due to inconsistencies in app usage and reporting, which varied widely across participants. In contrast, TFV-DP provides an objective biomarker of cumulative adherence over several weeks, and self-report offers a retrospective summary measure when biomarker data are unavailable. This dual approach allowed us to capture a more complete picture of adherence while reducing potential bias from missing or irregular app data. While the study team recognizes the value of real-time app data, the priority was to develop an adherence outcome that maximized both validity and data availability across the sample.

In summary, participants were classified as adherent to PrEP if their TFV-DP levels indicated usage of more than 4 pills per week, and as nonadherent if levels indicated fewer than 4 pills per week. For participants without TFV-DP measurements, self-reported survey data were used to minimize missingness. Those who reported taking PrEP at least 60% of the time were categorized as adherent, while those reporting less than 60% adherence were considered nonadherent.

#### Engagement

Engagement in P3 was defined as using at least 1 of the 3 key app features each day: medication tracker, social wall, or daily quest. Financial incentives, based on behavioral economic principles, were awarded [24,25]. Participants who interacted with any of the 3 features received US $0.50 deposited in their virtual bank account each day; those who did not interact had US $1.00 deducted. Virtual accounts were seeded with US $90 at the start of the study. Thus, participants who used the app every day for the entire 90-day study could amass US $135 at study completion; those who did not use the app at all would end the study with US $0. Three classifications of use were defined a priori based on financial incentives awarded at the end of the intervention: high use (>US $90 correlating to usage on >60 d), moderate use (US $67-$89.50 correlating to usage on 45‐59 d), and low use (<US $67 correlating to usage on <44 d). These values were chosen to align with prior studies of app engagement and literature regarding how long it takes to form a habit [16,30]. Additional paradata were collected, including total app log-ins, total days logged in, total time spent in the app (min), and total number of days participants tracked their PrEP use (taken or not taken). For those in the P3+ arm, the total number of in-app counseling sessions was also recorded.

#### Acceptability

Participants were asked acceptability-related questions at the 3 months. Three questions (eg, “I trust the information in P3,” “The information in P3 is easy to understand,” and “The information in P3 is accurate”) were adapted from Horvath et al [31] to assess end users’ perceptions of the quality of the information contained in the P3 app. These questions were assessed using a 4-point Likert scale, ranging from “strongly disagree” to “strongly agree,” where a higher score indicated better information quality. Then a dichotomized measure was created for each question, where responses of “agree” and “strongly agree” were interpreted as indicating high acceptability.

An adapted version (eg, questions altered slightly to align with mobile app intervention) of the Client Satisfaction Questionnaire-8 which included 8 items (eg, “How would you rate the quality of the help you have received from P3?” “Did you get the kind of help and/or support you wanted from P3?” “To what extent has P3 met your needs related to improving taking your meds?”) rated on 4-point Likert scale was used to assess satisfaction with the app [32]. The scale scores were computed by summing the responses to the 8 items (range 8‐32) with a higher score indicating a higher level of satisfaction (*α*=.89).

Two questions assessed participants’ likelihood of future use and willingness to pay to use the app. The first item, “I would use P3 in the future if available” was rated on a 4-point Likert scale (“strongly disagree” to “strongly agree”), with higher scores indicating higher likelihood of use. Then, a dichotomized measure was created, where responses of “agree” and “strongly agree” were interpreted as an intention to use P3 in the future if it becomes available. The second item, “If P3 were available in the app store, how much would you be willing to pay for it?” Response options included “US $0.99,” “US $1.99,” “US $2.99,” “more than $2.99,” and “I would not be willing to pay for it.” Responses were dichotomized into willing and unwilling to pay.

### Statistical Analysis

#### Engagement Analysis

Descriptive statistics for study variables at baseline were calculated. To examine the differences in intervention engagement and acceptability by arm, independent samples 2-tailed *t* tests for continuous variables and a chi-square analysis for categorical variables were conducted. To examine the effect of arm and engagement categories on PrEP adherence at 3 months, three logistic regression analyses were conducted: (1) the effect of arm on PrEP adherence, (2) the effect of engagement categories (high vs moderate or low) on PrEP adherence, and (3) the interaction effect of arm and engagement categories on PrEP adherence, along with the main effects of arm and engagement categories. We also included a single demographic variable in each of the 3 models to adjust for potential confounding effects. Due to imbalanced data, dichotomized engagement categories were used instead of 3 engagement categories. Finally, adherence data, including TFV-DP levels and self-reported adherence, were collected at a single time point during the 3-month follow-up visit, and thus the data are cross-sectional in nature. As such, analyses were conducted using cross-sectional methods, and any inferences about adherence patterns are based on a snapshot of behavior at that specific time.

#### Costing Analysis

The study team adopted a provider perspective and estimated the costs of P3/P3+ implementation in a community setting. This included the use of activity-based costing and, based on staff input, created an inventory of tasks associated with the management and implementation of P3 and P3+ [33]. The study team developed a survey in Qualtrics whereby each staff person at all sites reported time spent per week on the itemized task for a 2-week period. The survey was administered 4 times: October 2019, December 2019, February 2020, and November 2020 (post-COVID).

Cost per task was calculated by multiplying the median wage for the job category of the respondent staff member by the time spent on the activity. Costs per activity across staff were summed; extrapolated a monthly cost per activity based on the 2-week survey values and then averaged the monthly values by site and for all sites. Although each site provided enrollees for P3+, costs for P3+ were based on data from the North Carolina site only. Costs for P3 were based on all sites. The number of participants per month was estimated and based on the number enrolled in a given month, along with those participating in the intervention from the prior 2 months. Then, the average cost per enrollee per month was calculated. Costs for P3 app development were not included. Activities and materials specifically associated with grant requirements, such as laboratory tests, were not included in the calculations. The incremental cost-effectiveness ratio (ICER) was also calculated with respect to PrEP adherence and engagement in terms of number of app sessions, frequency, and time of app use (intensity), where the ICER is calculated as (Cost_P3+_ – Cost_P3_)/(Outcome_P3+_ – Outcome_P3_) [34].

### Ethical Considerations

The ATN P3 protocol, informed assent or consent documents, and related modifications were reviewed and approved by the University of North Carolina at Chapel Hill Institutional Review Board (IRB number 17‐1951). Informed consent was obtained from all participants, and no study activities were completed prior to giving informed consent. A waiver of parental consent to participate in this research study for youth participants aged 16 to 17 years was granted by the University of North Carolina at Chapel Hill Institutional Review Board. All data and records were stored on password-protected servers. This study is registered at ClinicalTrials.gov (NCT03320512). Participants were incentivized to use the P3 app and started with a baseline amount of US $ in a virtual bank account (US $90) and gained (+US $0.50) or lost (–US $1.00) money from this initial amount each day corresponding to use and nonuse of the app, respectively. The final amount in the virtual bank account (total possible ranged from US $0.00, representing no app use, to US $135, representing perfect daily app use) was paid out as part of the study remuneration at the 3-month study follow-up. Participants were compensated with US $50 upon completion of their enrollment visit, and US $50 each at the 3- and 6-month visits (US $150 total). Participants also received US $50 for each remote specimen collection. All study data were deidentified prior to analysis.

## Results

### Study Participant Demographic Characteristics

A total of 163 participants were randomized to the P3 intervention (n=82, 50% to the P3 arm; n=81, 50% to the P3+ arm). Retention was 84% (n=69) and 89% (n=72) at 3 months in the P3 and P3+ arms, respectively. The mean age of participants was 21.4 (SD 2.0) years, 91.4% (n=149) identified as male, 52% (n=85) identified as non-White, and 27% (n=44) of the sample identified as Hispanic. Of the total sample, 150 (92%) participants were actively taking PrEP at the time of the study, 6 (3.7%) participants were preparing to initiate PrEP for the first time, and 7 (4.3%) participants had recently resumed PrEP after a prior discontinuation. There were no significant differences in these descriptive statistics at baseline between the P3 and P3+ arms (Table 1).

**Table 1.** Descriptive statistics for study variables at baseline by intervention arm (N=163).

Variables	Total (N=163)	Arm		*P* value
		P3 intervention (n=82)	P3+ intervention (n=81)	
Age (y), mean (SD)	21.4 (2.0)	21.5 (2.0)	21.4 (2.1)	.74
Ethnicity, n (%)				.17
Hispanic	44 (27)	26 (59.1)	18 (40.9)	
Non-Hispanic	119 (73)	56 (47.1)	63 (52.9)	
Race				.31
Others	85 (52.2)	46 (56.1)	39 (48.1)	
White	78 (47.8)	36 (43.9)	42 (51.9)	
Currently attending school				.45
No	59 (36.2)	32 (54.2)	27 (45.8)	
Yes	104 (63.8)	50 (48.1)	54 (51.9)	
Currently employed				.58
No	41 (25.2)	20 (48.8)	21 (51.2)	
Yes	121 (74.2)	62 (51.2)	59 (48.8)	
Gender identity				.09
Man	149 (91.4)	78 (52.3)	71 (47.7)	
Other	14 (8.6)	4 (28.6)	10 (71.4)	

### Intervention Engagement and Acceptability

Overall engagement was high in P3+ arm compared to the P3 arm (Table 2). Participants in the P3+ arm earned higher incentives ($90.6 vs $75.4; *P*=.04), had more app log-ins (96.6 vs 76.1; *P*=.01), used the app on more days (63.3 d vs 53.2 d; *P*=.04), and spent more time in the intervention (378.8 min vs 186.66 min; *P*<.001) compared to those in the P3 arm. Overall acceptability was high in both study arms, and there was no significant difference between P3 and P3+ arms.

**Table 2.** Intervention engagement and acceptability at 3 months (N=163).

Variables	Total (N=163)	Arm		*P* value
		P3 intervention (n=82)	P3+ intervention (n=81)	
Engagement				
Total incentives earned (US $), mean (SD)	83.0 (46.6)	75.4 (48.4)	90.6 (43.6)	.04
Engagement category, n (%)				.15
High	95 (58.3)	42 (51.2)	53 (65.4)	
Moderate	12 (7.4)	6 (7.3)	6 (7.4)	
Low	56 (34.3)	34 (41.5)	22 (27.2)	
Number of sessions, mean (SD)	86.3 (52.6)	76.1 (51.0)	96.6 (52.5)	.01
Days used, mean (SD)	58.2 (31.1)	53.2 (32.1)	63.3 (29.3)	.04
Time spent in app (min), mean (SD)	282.1 (242.0)	186.6 (166.5)	378.8 (267.9)	<.001
Days PrEP^a^ tracked, mean (SD)	65.9 (33.5)	61.7 (34.7)	70.1 (31.9)	.11
Coaching sessions attended, mean (SD)	—^b^	—	3.50 (1.6)	—
Acceptability				
Information quality, n (%)				
Trust information	132 (98.5)	67 (100)	65 (97)	.15
Information easy to understand	132 (98.5)	67 (100)	65 (97)	.15
Information is accurate	130 (97)	66 (98.5)	64 (95.5)	.31
Satisfaction (CSQ-8)^c^, mean (SD)	24.7 (5.0)	24.4 (4.9)	25.0 (5.0)	.50
Future use, n (%)				
Use in future if available	97 (72.4)	50 (73.5)	47 (71.2)	.76
Willingness to pay	52 (38.5)	25 (36.8)	27 (40.3)	.67

a PrEP: pre-exposure prophylaxis.

b Not applicable.

c CSQ-8: Client Satisfaction Questionnaire-8.

### The Effect of Intervention Arm and Engagement on PrEP Adherence

Using the composite endpoint which included both self-report and TFV-DP levels, adherence was 78.6% (n=55) in the P3 arm and 78.7% (n=59) in the P3+ arm. As shown in Table 3, there was no significant association between intervention arm and PrEP adherence at 3 months (*P*=.989). Engagement category (high vs moderate or low) was significantly associated with PrEP adherence at 3 months (*P*=.003); however, the interaction effect between intervention status and engagement categories on PrEP adherence was not significant (*P*=.577). We tested models including a single demographic variable to adjust for potential confounding, but the results remained unchanged (Table S1 in Multimedia Appendix 1).

**Table 3.** Relationship between intervention arm and engagement categories on pre-exposure prophylaxis (PrEP) adherence at 3 months.

	PrEP adherence^a^		
	Estimate	SE	*P* value
Intervention arm			
P3+ intervention vs P3 intervention	0.01	0.40	.99
Engagement category			
High vs moderate/low	1.27	0.42	.003
Interaction between arm and engagement category			
P3+ intervention vs P3 intervention	−0.38	0.59	.52
High vs moderate/low	1.04	0.60	.08
P3+ intervention vs P3 intervention and high vs moderate/low	0.47	0.84	.58

a Composite outcome.

### Intervention Cost

Table S2 in Multimedia Appendix 1 shows the average monthly costs per activity. Intervention implementation and management costs were, on average, US $617 (SD $270.9) per month across sites with staff meetings accounting for over half of the monthly total. Average monthly costs associated with participant interactions were US $502 (SD $78.6). Participant recruitment, at US $204 (SD $100.0), was the primary cost driver, accounting for 41% of the total, and the largest reported expense across sites. Troubleshooting was the second with Chapel Hill, the central study site, reporting relatively higher expense than other sites.

Taken together, the overall average total monthly cost of P3 was US $1118 (SD $305.1) and ranged from US $797 to $1368. Sites averaged 4.5 (SD 3.9) participants per month resulting in an average total monthly cost per participant of US $280 (SD $118.5). Table S3 in Multimedia Appendix 1 shows the additional costs associated with the P3+ intervention. Average monthly costs for ongoing management of P3+ were US $655 (SD $263.0), while those for counseling-related issues were US $565 (SD $45.0), dominated by counseling itself. An estimated 2.3 participants received counseling per month at an average cost of US $72 (2.7).

Given the difference in P3+ and P3 adherence was small and not significant, the ICER was not calculated. The ICER for the number of app sessions was US $7.06 ([US $352-US $280]/[US $86.3‐US $76.1]) per additional session used, while the ICER for time in session was US $45 per additional hour of use.

## Discussion

The findings from this analysis underscore the critical role that user engagement plays in the effectiveness of DHIs aimed at enhancing adherence to PrEP among young individuals at high risk for HIV. A key finding from this study is the significant association between higher levels of engagement with the P3 intervention and increased adherence to PrEP. This aligns with prior research, which has consistently demonstrated that user engagement is a pivotal factor in the success of DHIs across various health domains, including HIV care and prevention [13,14]. Despite the high levels of engagement observed in this study, it is challenging to draw direct comparisons with other DHIs due to the lack of standardized metrics for measuring engagement. A recent scoping review focused on engagement in DHIs for sexual and gender minorities noted that only a small fraction of trials reported engagement metrics, and even fewer studies defined an “optimal” level of engagement necessary to achieve significant health outcomes [35].

The P3 app employed several strategies to boost user engagement, including financial incentives and gamification [19,23]. These methods are supported by a growing body of evidence that suggests such approaches can effectively maintain user interaction over extended periods. For example, Looyestyn et al [20] conducted a systematic review that concluded gamification often increases engagement with online health programs, although the long-term effects on health outcomes remain uncertain. However, enhancing engagement strategies through more robust and personalized approaches has the potential to further improve adherence rates [36]. Moreover, conducting longer-term studies could offer valuable insights into the lasting impact of these strategies, thereby enabling the refinement and adaptation of interventions for sustained long-term outcomes [37].

Interestingly, this study observed that while the P3+ intervention, which included in-app adherence coaching, led to increased engagement (eg, more time spent in the app and higher incentive earnings), this did not translate into significantly better adherence compared to the standard P3 arm. This, coupled with the increased cost and complexity of P3+ delivery, indicates that further studies are necessary to determine whether this intensified intervention is the appropriate fit. This finding may indicate a potential ceiling effect where additional engagement beyond a certain threshold does not necessarily result in proportional improvements in adherence. Using sequential multiple assignment randomized trials could be particularly useful in this context, as they would allow for the systematic evaluation and optimization of the intervention’s components (and human resources) to better tailor the approach to individual needs [35]. Our results here show that increasing frequency of use was of relatively low cost, while increasing intensity of use cost US $45 an hour. As these are interim outcomes, we cannot declare whether they are cost-effective as we have no standard against which to measure the results as the literature in this area is extremely limited. Although the P3+ compared ot P3 did not increase adherence, it may have produced other health benefits that were not measured as part of the trial.

Given the challenges of sustaining engagement in standalone digital health interventions, future research may explore whether adherence support features could be effectively delivered through widely used social and dating platforms. These platforms attract substantial daily engagement from populations at high risk for HIV, suggesting potential reach. However, our study did not collect data on participants’ platform preferences, usage patterns, or privacy concerns, and thus any discussion of integrating interventions into these environments remains hypothetical. Importantly, any such approach would require additional research to assess user acceptability, data security considerations, and potential risks, as well as partnerships with platform developers. If found feasible and acceptable, embedding adherence support within high-traffic commercial apps could reduce the costs of sustaining standalone interventions and may leverage existing infrastructure for personalization and scalability.

This study has several limitations that must be acknowledged. Collection of biologic outcome data was impacted by the COVID-19 pandemic, potentially affecting the accuracy of adherence measurements and the robustness of the findings. The cost analysis presented is based on data collected over a limited period, with potential disruptions also caused by the COVID-19 pandemic. Additionally, due to the small number of participants who were initiating PrEP for the first time, the study team was unable to conduct meaningful comparisons between novice and experienced PrEP users. This limited the ability to explore whether patterns of adherence or engagement differed by prior PrEP experience. Future studies with a more balanced distribution of new and experienced users are needed to examine these potential differences. Moreover, this study was unable to adjust for behavioral confounders such as sexual activity frequency, PrEP experience, and partner status, as well as demographic confounders that are known to influence the relationship between engagement and adherence. This may result in an estimated association that reflects the influence of confounders rather than the true effect of the exposure. Due to the limited sample size, we were unable to explicitly examine how these confounders affect engagement and adherence, or how they interact with one another. Future research with larger samples is warranted to enable comprehensive adjustment for these factors and to better understand their combined impact. Furthermore, variability in site-specific factors, such as participant recruitment and staff composition, likely contributed to the wide range of cost estimates. While staff were asked to report time for non–study-related tasks, separating the two may have been challenging, particularly in relation to staff meetings. In addition, some tasks conducted in a study setting, such as recruitment, may not be a good proxy for real-world costs and are likely an overestimate. A notable limitation of this cost analysis is the exclusion of initial app development expenses, which are often among the most substantial costs for digital health interventions. This decision was made to provide a clearer estimate of the ongoing operational and maintenance costs associated with scaling the P3 and P3+ interventions in community settings, as these are the costs most likely to be absorbed by public health agencies or clinical partners. Including development costs would significantly increase the apparent total cost, potentially skewing the analysis away from the more immediate, recurring expenses that directly impact long-term sustainability. Moreover, app development costs can vary widely based on factors such as platform selection (eg, proprietary vs open-source), feature complexity, integration with existing health care systems, and ongoing user feedback. For instance, the P3 app incorporated multiple interactive elements, including social walls, daily quests, and personalized adherence coaching, each of which adds to the initial investment. Given this variability, including these costs would complicate direct cost comparisons across different digital health interventions. Nonetheless, future studies should consider a more comprehensive economic evaluation that includes both fixed and variable costs, as this will be critical for assessing the true scalability and long-term viability of such digital health solutions in real-world settings. Finally, this study provided financial incentives for app engagement which may not be sustainable outside of research contexts.

In conclusion, this study highlights the critical role of user engagement in enhancing PrEP adherence among young individuals at high risk for HIV. While increased engagement through DHIs like the P3 app correlates with higher adherence rates, the findings suggest that simply intensifying interventions, such as with the P3+ model, may not necessarily lead to proportionate improvements. The additional costs and complexities associated with more intensive approaches require careful consideration and further investigation. Future research should prioritize the development of more personalized and adaptive engagement strategies, leveraging methodologies such as sequential multiple assignment randomized trials designs to optimize intervention components in real-time. By focusing on long-term adherence and the sustainability of engagement, these efforts can better inform the design of scalable, cost-effective digital health solutions that are both effective and responsive to the evolving needs of at-risk populations.

## Supplementary material

10.2196/66837 Multimedia Appendix 1 Relationship between intervention arm and engagement categories on PrEP adherence at 3 months, adjusted for demographics.

## Abbreviations

ATN: Adolescent Medicine Trials Network for HIV Interventions
DHI: digital health intervention
ICER: incremental cost-effectiveness ratio
P3: Prepared, Protected, emPowered
PrEP: pre-exposure prophylaxis
TFV-DP: tenofovir-diphosphate

## References

[1] Grant RM, Lama JR, Anderson PL (2010). Preexposure chemoprophylaxis for HIV prevention in men who have sex with men. N Engl J Med.

[2] Anderson PL, Glidden DV, Liu A (2012). Emtricitabine-tenofovir concentrations and pre-exposure prophylaxis efficacy in men who have sex with men. Sci Transl Med.

[3] Hosek SG, Landovitz RJ, Kapogiannis B (2017). Safety and feasibility of antiretroviral preexposure prophylaxis for adolescent men who have sex with men aged 15 to 17 years in the United States. JAMA Pediatr.

[4] Hosek S, Celum C, Wilson CM, Kapogiannis B, Delany‐Moretlwe S, Bekker L (2016). Preventing HIV among adolescents with oral PrEP: observations and challenges in the United States and South Africa. J Int AIDS Soc.

[5] Cambou MC, Landovitz RJ (2021). Challenges and opportunities for preexposure prophylaxis. Top Antivir Med.

[6] Ezennia O, Geter A, Smith DK (2019). The PrEP care continuum and Black men who have sex with men: a scoping review of published data on awareness, uptake, adherence, and retention in PrEP care. AIDS Behav.

[7] Sun L, Qu M, Chen B, Li C, Fan H, Zhao Y (2023). Effectiveness of mHealth on adherence to antiretroviral therapy in patients living with HIV: meta-analysis of randomized controlled trials. JMIR Mhealth Uhealth.

[8] Bauermeister J, Choi SK, Bruehlman-Senecal E (2022). An identity-affirming web application to help sexual and gender minority youth cope with minority stress: pilot randomized controlled trial. J Med Internet Res.

[9] Forbes A, Keleher MR, Venditto M, DiBiasi F (2023). Assessing patient adherence to and engagement with digital interventions for depression in clinical trials: systematic literature review. J Med Internet Res.

[10] Regmi K, Kassim N, Ahmad N, Tuah NA (2017). Effectiveness of mobile apps for smoking cessation: a review. Tob Prev Cessat.

[11] Couper MP, Alexander GL, Zhang N (2010). Engagement and retention: measuring breadth and depth of participant use of an online intervention. J Med Internet Res.

[12] Choi SK, Golinkoff J, Michna M, Connochie D, Bauermeister J (2022). Correlates of engagement within an online HIV prevention intervention for single young men who have sex with men: randomized controlled trial. JMIR Public Health Surveill.

[13] Hightow-Weidman LB, LeGrand S, Muessig KE (2019). A randomized trial of an online risk reduction intervention for young Black MSM. AIDS Behav.

[14] Hightow-Weidman L, Muessig KE, Egger JR, Vecchio A, Platt A (2021). Epic allies: a gamified mobile app to improve engagement in HIV care and antiretroviral adherence among young men who have sex with men. AIDS Behav.

[15] Fan X, Ning K, Liu C (2023). Uptake of an app-based case management service for HIV-positive men who have sex with men in China: process evaluation study. J Med Internet Res.

[16] Hightow-Weidman LB, Bauermeister JA (2020). Engagement in mHealth behavioral interventions for HIV prevention and care: making sense of the metrics. Mhealth.

[17] Hightow-Weidman LB, Horvath KJ, Scott H, Hill-Rorie J, Bauermeister JA (2021). Engaging youth in mHealth: what works and how can we be sure?. Mhealth.

[18] Hightow-Weidman L, Muessig K, Knudtson K (2018). A gamified smartphone app to support engagement in care and medication adherence for HIV-positive young men who have sex with men (AllyQuest): development and pilot study. JMIR Public Health Surveill.

[19] Tran S, Smith L, El-Den S, Carter S (2022). The use of gamification and incentives in mobile health apps to improve medication adherence: scoping review. JMIR Mhealth Uhealth.

[20] Looyestyn J, Kernot J, Boshoff K, Ryan J, Edney S, Maher C (2017). Does gamification increase engagement with online programs? A systematic review. PLoS ONE.

[21] Sardi L, Idri A, Fernández-Alemán JL (2017). A systematic review of gamification in e-Health. J Biomed Inform.

[22] Cugelman B (2013). Gamification: what it is and why it matters to digital health behavior change developers. JMIR Serious Games.

[23] LeGrand S, Knudtson K, Benkeser D (2018). Testing the efficacy of a social networking gamification app to improve pre-exposure prophylaxis adherence (P3: Prepared, Protected, emPowered): protocol for a randomized controlled trial. JMIR Res Protoc.

[24] Kimmel SE, Troxel AB (2012). Novel incentive-based approaches to adherence. Clin Trials.

[25] Haff N, Patel MS, Lim R (2015). The role of behavioral economic incentive design and demographic characteristics in financial incentive-based approaches to changing health behaviors: a meta-analysis. Am J Health Promot.

[26] Vlaev I, King D, Darzi A, Dolan P (2019). Changing health behaviors using financial incentives: a review from behavioral economics. BMC Public Health.

[27] Amico KR, Miller J, Balthazar C (2019). Integrated next step counseling (iNSC) for sexual health and PrEP use among young men who have sex with men: implementation and observations from ATN110/113. AIDS Behav.

[28] Blumenthal J, Pasipanodya EC, Jain S (2019). Comparing self-report pre-exposure prophylaxis adherence questions to pharmacologic measures of recent and cumulative pre-exposure prophylaxis exposure. Front Pharmacol.

[29] Zimmerman RS, Mehrotra P, Madden T, Paul R (2021). The value of assessing self-reported and biological indicators of outcomes in evaluating HIV programs. Curr HIV/AIDS Rep.

[30] Lally P, van Jaarsveld CHM, Potts HWW, Wardle J (2010). How are habits formed: Modelling habit formation in the real world. Euro J Social Psych.

[31] Horvath KJ, Oakes JM, Rosser BRS (2013). Feasibility, acceptability and preliminary efficacy of an online peer-to-peer social support ART adherence intervention. AIDS Behav.

[32] Kelly PJ, Kyngdon F, Ingram I, Deane FP, Baker AL, Osborne BA (2018). The Client Satisfaction Questionnaire-8: Psychometric properties in a cross-sectional survey of people attending residential substance abuse treatment. Drug Alcohol Rev.

[33] Drummond MF, Sculpher MJ, Torrance GW, O’Brien BJ, Stoddart GL (2002). Methods for the Economic Evaluation of Health Care Programmes.

[34] Brandão SMG, Brunner-La Rocca HP, de Lima ACP, Bocchi EA (2023). A review of cost-effectiveness analysis: from theory to clinical practice. Medicine (Baltimore).

[35] Choi SK, Muessig KE, Hightow-Weidman LB, Bauermeister JA (2023). Paradata: measuring engagement in digital HIV interventions for sexual and gender minorities. Curr HIV/AIDS Rep.

[36] Saleem M, Kühne L, De Santis KK, Christianson L, Brand T, Busse H (2021). Understanding engagement strategies in digital interventions for mental health promotion: scoping review. JMIR Ment Health.

[37] Nahum-Shani I, Smith SN, Spring BJ (2018). Just-in-time adaptive interventions (JITAIs) in mobile health: key components and design principles for ongoing health behavior support. Ann Behav Med.

