# Supplementary material for: Improving Pre-Exposure Prophylaxis Adherence in People at Risk for HIV: Secondary Analysis of a Digital Health Intervention to Enhance User Engagement
Source: JMIR Mhealth Uhealth. 2026 Mar 10;14:e66837. doi: 10.2196/66837 (PMC12974996; doi:10.2196/66837)
Supplement: Multimedia Appendix 1 [file mhealth-v14-e66837-s001.docx]

**Supplemental Table 1: Relationship between Intervention Arm and Engagement Categories on PrEP Adherence at 3-month, adjusted for demographics.**

|  |  |  | **PrEP Adherence**  **^Estimate (SE), p-value^** | | | | | | |
| --- | --- | --- | --- | --- | --- | --- | --- | --- | --- |
|  |  |  | Null Model | Model 1:  Age | Model 2: Ethnicity | Model 3:  Race | Model 4: Attending School | Model 5:  Employment | Model 6:  Gender identity |
| Intervention arm | | | | | |  |  |  |  |
|  | P3+ Intervention vs. P3 Intervention |  | 0.01 (0.40),  .989 | 0.003 (0.41),  .994 | 0.008 (0.41),  .984 | -0.06 (0.42),  .892 | -0.000 (0.41),  .999 | -0.01 (0.41),  .989 | -0.09 (0.41),  .836 |
| Engagement category | | | | | |  |  |  |  |
|  | High vs. Moderate/Low |  | 1.27 (0.42),  **.003** | 1.22 (0.42),  **0.004** | 1.28 (0.42),  **.002** | 1.13 (0.43),  **.009** | 1.28 (0.43),  **.003** | 1.34 (0.43),  **.002** | 1.31 (0.42),  **.002** |
| Interaction between arm and engagement category | | | | | |  |  |  |  |
|  | P3+ Intervention vs. P3 Intervention |  | -0.38 (0.59),  .522 | -0.36 (0.60),  .548 | -0.37 (0.59),  .538 | -0.47 (0.62),  .448 | -0.38 (0.60),  .523 | -0.33 (0.62),  .595 | -0.57 (0.61),  .356 |
|  | High vs. Moderate/Low |  | 1.04 (0.60),  .081 | 1.02 (0.60),  .091 | 1.06 (0.60),  .078 | 0.88 (0.61),  .153 | 1.06 (0.60),  .079 | 1.18 (0.62),  .057 | 1.06 (0.60),  .077 |
|  | P3+ Intervention vs. P3 Intervention | High vs. Moderate/Low | 0.47 (0.84),  .577 | 0.44 (0.85),  .600 | 0.47 (0.84),  .575 | 0.54 (0.87),  .534 | 0.46 (0.85),  .585 | 0.34 (0.88),  .698 | 0.57 (0.85),  .501 |

Note. Model 1 adjusted for age (continuous). Model 2 adjusted for ethnicity (non-Hispanic vs Hispanic). Model 3 adjusted for race (Other race vs White). Model 4 adjusted for attending school currently (No vs Yes). Model 5 adjusted for employment status (Not employed vs currently employed). Model 6 adjusted for gender identity (Man vs other gender identity).

**Supplemental Table 2: Average Monthly P3 Cost per Activity and Participant by Site**

| Site | Atlanta | Boston | Chapel  Hill | Chicago | Houston^a^ | Philadelphia | Tampa | All  Sites |
| --- | --- | --- | --- | --- | --- | --- | --- | --- |
| Activity | Cost $ (s.d.) | Cost $ (s.d.) | Cost $ (s.d.) | Cost $ (s.d.) | Cost $  (s.d.) | Cost $  (s.d.) | Cost $  (s.d.) | Cost $  (s.d.) |
| **I. Intervention Implementation and On-Going Management** |  |  |  |  |  |  |  |  |
| Hiring staff for P3 implementation | 83  (6.9) | 35  (15.0) | 242  (58.2) | 0 | 88  (29.8) | 26  (13.2) | 13  (5.7) | 70  (76.9) |
| Staff meetings related to P3 | 250  (22.6) | 118  (9.2) | 523  (43.9) | 325  (67.4) | 725  (211.5) | 180  (20.8) | 290  (15.1) | 344  (195.4) |
| Development of training materials | 42  (1.9) | 95  (41.1) | 32  (10.5) | 0 | 26  (7.1) | 57  (28.5) | 48  (12.7) | 43  (27.2) |
| Staff Initial/refresher training | 65  (2.9) | 101  (40.3) | 96  (25.3) | 48  (20.9) | 43  (10.8) | 4  (2.2) | 59  (25.8) | 60  (30.4) |
| Other activities | 71  (6.8) | 36  (9.8) | 144  (34.1) | 346  (125.6) | 69  (32.7) | 0 | 39  (6.5) | 101  (108.1) |
| Total | 512  (23.4) | 384  (82.2) | 1037  (53.0) | 719  (213.0) | 952  (217.0) | 268  (45.7) | 450  (47.0) | 617  (270.9) |
| **II. Interaction with Participants** |  |  |  |  |  |  |  |  |
| Participant Recruitment | 184  (20.9) | 320  (105.2) | 97  (11.4) | 381  (105.8) | 160  (4.8) | 185  (48.1) | 99  (23.4) | 204  (100.0) |
| Participant Enrollment | 43  (12.3) | 108  (27.1) | 41  (12.8) | 45  (14.8) | 112  (29.1) | 0 | 195  (50.6) | 78  (60.4) |
| On-going P3 management and  troubleshooting | 101  (22.5) | 36  (5.1) | 384  (19.5) | 57  (24.8) | 61  (17.8) | 250  (53.9) | 67  (15.1) | 136  (121.0) |
| Assistance with P3 issues post  enrollment | 55  (10.6) | 0 | 17  (7.3) | 9  (2.3) | 24  (5.6) | 17  (8.6) | 26  (9.7) | 21  (16.2) |
| Ordering/following up on mail in  blood kits | 7  (3.2) | 0 | 14  (6.1) | 5  (2.0) | 0 | 4  (2.2) | 0 | 4  (4.8) |
| Other activities | 38  (10.4) | 17  (7.4) | 64  (18.3) | 112  (17.0) | 59  (5.6) | 72  (20.9) | 48  (12.0) | 59  (27.7) |
| Total | 428  (18.1) | 481  (129.3 | 617  (20.5) | 609  (98.1) | 416  (37.1) | 529  (107.0) | 435  (92.7) | 502  (78.6) |
|  |  |  |  |  |  |  |  |  |
| Total | 940  (36.7) | 864  (148.8) | 1654  (55.2) | 1328  (305.9) | 1368  (181.2) | 797  (88.7) | 875  (139.6) | 1118  (305.1) |
|  |  |  |  |  |  |  |  |  |
| Average number of participants per month^b^ | 3.75  (4.82) | 4.75  (5.95) | 2.5  (3.79) | 5.25  (6.41) | 4.5  (6.01) | 5  (6.32) | 5.75  (7.03) | 4.5  (3.90) |
| Average total monthly cost per participant^c^ | 251 | 182 | 661 | 253 | 304 | 159 | 152 | 280  (118.5) |

^a^ Used three months of data for Houston

^b^ Approximation given that enrollment was rolling, and participants remained in the project for up to six months

^c^ Did not calculate standard deviations around total monthly costs per participant as some months had zero participants leading to undefined values.

**Supplemental Table 3: Average Monthly P3+ Cost per Activity and Participant**

| Activity^a^ | Cost $  (s.d.) |
| --- | --- |
| Intervention Evaluation |  |
| Monitoring use of P3+ features by participants | 214  (27.3) |
| Data processing related to P3+ use/engagement | 349  (35.8) |
| Other activities | 92  (38.9) |
| Total | 655  (263.0) |
|  | |
| Counseling |  |
| Administration of counseling | 46  (4.5) |
| Counseling participants | 340  (34.3) |
| Case conferences about counseling | 26  (4.4) |
| Data management | 153  (12.8) |
| Total | (565)  (45.0) |
|  | |
| Average total monthly cost | 1220  (60.0) |
| Average number of participants per month | 17  (0.91) |
| Average total monthly cost per participant | 72  (2.7) |

^a^ Enrollment took place across sites, while the P3+ intervention was implemented by Chapel Hill.
